# Recent Advances in MEMS Metasurfaces and Their Applications on Tunable Lens

**DOI:** 10.3390/mi10080505

**Published:** 2019-07-31

**Authors:** Shaowei He, Huimin Yang, Yunhui Jiang, Wenjun Deng, Weiming Zhu

**Affiliations:** School of Optoelectronic Science and Engineering, University of Electronic Science and Technology of China, Chengdu 610054, China

**Keywords:** MEMS metasurface, tunable lens, microfluidic metasurface, structural reconfiguration

## Abstract

The electromagnetic (EM) properties of metasurfaces depend on both structural design and material properties. microelectromechanical systems (MEMS) technology offers an approach for tuning metasurface EM properties by structural reconfiguration. In the past 10 years, vast applications have been demonstrated based on MEMS metasurfaces, which proved to have merits including, large tunability, fast speed, small size, light weight, capability of dense integration, and compatibility of cost-effective fabrication process. Here, recent advances in MEMS metasurface applications are reviewed and categorized based on the tuning mechanisms, operation band and tuning speed. As an example, the pros and cons of MEMS metasurfaces for tunable lens applications are discussed and compared with traditional tunable lens technologies followed by the summary and outlook.

## 1. Introduction

Metasurfaces are planar artificial materials consisting of subwavelength structures, which are also known as meta-atoms, with engineered electromagnetic properties. The subwavelength structures, which are typically far thinner than the working wavelength, can abruptly change the phase, amplitude, and polarization state of the incident light, making metasurfaces possible for vast applications including filter [1,2], active color control [3], switch [4,5], perfect absorber [6,7,8,9], and polarizer [10,11], etc. Governed by Generalized Snell’s Law [12], metasurfaces can tailor the wavefront of the incident EM waves by spatially varying the localized phase change, which lead to applications such as beam steering [13,14], flat lens [15,16,17,18,19,20,21], etc. Metasurface devices have merits such as compact sizes, easy in vertical integration and feasibility of mass fabrication etc. Enabled by recent development of micro- and nano-fabrication technologies, metasurfaces have now been demonstrated with working frequencies from GHz to visible region [22,23,24,25,26,27], which make them promising candidates for densely integrated planar optical components.

Driven by the demand of active and tunable photonic devices in the market, tunable and reconfigurable metasurfaces have attracted more and more research interest during the last decade [28,29,30,31]. The EM properties of metasurfaces depend on both structural design and material properties of meta-atoms, which naturally lead to two different approaches for the realization of tunable metasurfaces. Like many other photonic devices, changing material refractive indexes is a straight forward and effective way of making active metasurfaces, which is first demonstrated in THz region [32]. However, the selection of nonlinear materials is quite limited for active metasurface devices, which require a large amount of energy to trigger the nonlinear effect of the materials. Therefore, the tuning process of such devices are typically power consuming and it is very hard to maintain the optical performances with increasing ohmic losses. The tuning speed and range of active metasurfaces are bottlenecked by the nonlinear effects of the materials—e.g., free carrier effect [33,34], thermos-optical effect [35], etc. —which are highly dependent on the material properties. In most cases, materials with large nonlinear optical effects—e.g., gallium, liquid crystals, etc., —are excluded from densely integrated devices and cost-effective fabrication processes, which limit the practical applications of active metasurfaces. On the other hand, structural reconfiguration offers the other approach for making tunable metasurfaces via changing the structures or the arrangements of meta-atoms, which are called structural reconfigurable metasurfaces. The phase, polarization, and amplitude modulation of incident EM waves is dominated by the geometry of the metamolecules, which are highly sensitive to structural reconfiguration of the metasurfaces. The structural reconfigurations of metasurfaces can be realized using different methods, including stretching the flexible substrates [36,37,38,39,40,41], mechanical displacement of multi-layered metasurfaces [37,38], etc. However, the practical applications of structural reconfigurable metasurfaces are often humped by the size and the tuning speed of control systems, which have now been greatly improved by using MEMS technology [39,40,41,42,43,44,45,46,47,48,49,50,51]. The benefit of MEMS metasurfaces is twofold. One is the tuning speed depends on the Young’s modulus of the mechanical system and the actuation mass, which is not limited by the nonlinearity of the materials. Smaller actuation mass can, in theory, have larger tuning speed. The other one is that MEMS systems are intensively used in densely integrated devices and compatible to vast cost effective fabrication processes. Recently, microfluidic systems are also used in structural reconfigurable metasurfaces, which show individually control of meta-atoms [52].

Limited by current technologies of MEMS actuators, the tuning speed of MEMS metasurfaces are demonstrated up to KHz and capped at MHz, which are several orders slow than those of active metasurfaces—e.g., the typical relaxation time of free carrier injection effect is 10^−12^ s corresponding to THz tuning speed [53,54]. However, the freedom of materials choices and large tuning range make MEMS metasurfaces competitive in some applications where KHz to MHz modulation speed is acceptable—e.g., tunable lenses. The state-of-art technologies of tunable lenses are based on either changing the refractive index/geometry of the lenses or adjusting the relative positions of lenses in a group. It is very difficult for those tunable lenses to have fast tuning speed due to the large actuation mass of the lenses. The typical tuning speed of the tunable lenses available in the market is ranging from sub-hertz to tens of hertz, which is the bottleneck of the response time for many imaging and monitoring devices. Also, the performances of the lenses are not well maintained during the tuning process since the spatial resolution of refractive index/geometry tuning is far larger than the wavelength. The wavefront of incidence cannot be well controlled during the tuning process, which, as a result, increase the aberrations of the tunable lenses. Tunable lenses applications are also limited by their feasibilities of on-chip integration. One example is that most smartphone rely on software for the camera focusing. MEMS metasurfaces, on the other hand, are good candidates for tunable lens applications. In most cases, metasurfaces are subwavelength-thin structures with silicon substrate. The mechanical resonance frequency of such devices are very high (KHz to MHz) due to their small mass and large Young’s modulus of silicon. Therefore, the bottleneck of the tuning speed of MEMS metasurface lens can go up to KHz or even MHz depend on the mechanical design. The feasibility of densely integration is another merit of MEMS metasurfaces on tunable lens applications. Here, recent advances in MEMS metasurface applications are reviewed and categorized based on the tuning mechanisms in Section 2. The pros and cons of MEMS metasurfaces for tunable lens applications is discussed and compared with traditional tunable lens technologies in Section 3. Finally, the summary and outlook is given in Section 4.

## 2. Microelectromechanical Systems (MEMS) Metasurfaces

In the early stage of MEMS metasurface research, MEMS actuations are applied to single or a few meta-atoms or antennas to demonstrate the concept of structural reconfiguration in GHz [55]. However, MEMS actuators have been intensively studied for micrometer displacement [56,57], which can be used for structural reconfiguration of meta-atoms working at THz band with tens of micrometers in size.

**Figure 1:**
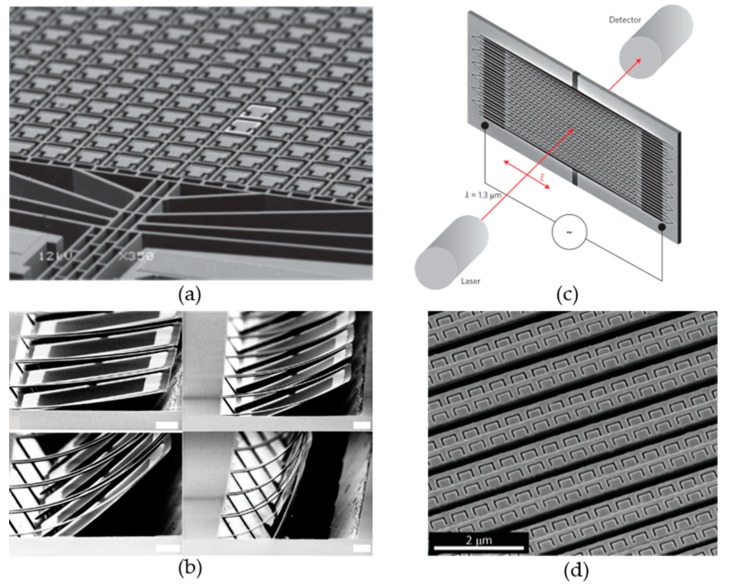
Microelectromechanical systems (MEMS) metasurfaces working at THz and infrared region. (**a**,**b**) optical switches driven by electrostatic force working at THz and infrared region, respectively; (**c**,**d**) amplitude modulators driven by thermo-mechanical actuation working at THz and infrared region, respectively. Figure 1a–d are reprinted with permission from [58] copyright by Wiley Online Library, 2011, [59] copyright by API Publishing, 2009, [60] copyright by Springer Nature, 2013, and [61] copyright by ACS, 2011, respectively.

Pioneer works on MEMS metasurfaces with meta-atoms array are demonstrated in THz region as shown in Figure 1a,b [58,59]. Figure 1a shows a MEMS metasurface driven by electrostatic force with metamolecules of 30 μm in size. The split ring metamolecules are composed of two identical half ring aluminum resonators. One is located on an isolated substrate with 10s μm in size. The other one is located on a frame substrate with a 3-μm width, which requires much less release time than the one on isolated substrate. Only the half rings located on frame substrate are released by controlling the release time. The frame substrate is connected to two MEMS comb drive actuators, which can be used to change the relative positions of the two half rings in each metamolecules. This work demonstrates tuning of the magnetic resonance in real time. Figure 1b shows another pioneer work on MEMS metasurface driven by thermo-actuators. The split ring structures can be flipped out of the metasurface plane and induce magnetic resonance with normal incidence. Those MEMS actuators change the geometry structures of metamolecules and tune their EM response, which is called inner-atom structural reconfiguration. As a result, the EM properties of the metasurfaces are controlled by MEMS actuators.

Later, MEMS metasurfaces working at infrared region [60,61] have been demonstrated as shown in Figure 1b,d. The working principle of MEMS actuators are similar to those of THz MEMS metasurfaces. However, it is very difficult to realize inner-atom structural reconfiguration due to the nanometer size of the infrared meta-atoms. The inner-atom actuations dramatically increase the fabrication difficulties. Therefore, MEMS actuators are used to control the couplings between infrared meta-atoms by changing their relative positions, which is called inter-atom structural reconfiguration. The lattice structure of infrared metasurfaces are tuned by shifting one row of metamolecules simultaneously. In this way, the EM properties of infrared metasurfaces are controlled by MEMS actuation. With the development of nanoelectromechanical systems (NEMS) [62], it can be expected that inner-atom structural reconfiguration of infrared metasurface will be demonstrated in the near future.

### 2.1. Dynamic Modulation of Frequency, Amplitude, and Polarization State

Both inner- and inter-atom structural reconfigurations can change the EM resonances within the meta-atoms, which modulate the frequency and amplitude of the incident EM waves. Figure 1 shows MEMS metasurfaces functioning as optical switches and variable optical attenuators realized by tuning the resonance frequencies. Besides resonance frequency tuning, polarization control is also a desire of many devices such as polarizer, optical trapping, beam formation, etc. From a material point of view, polarization control directly linked to the symmetry of the atoms or lattice constant of the materials. Artificial metamolecules can be designed to have desired symmetry according to their applications. However, it is very difficult for active metasurface to change the symmetry of their lattice or metamolecules. However, structural reconfiguration can be applied to change the symmetry of meta-atoms or lattices of the metasurfaces by tuning the geometry or relative positions of the metamolecules, which enables polarization control of EM waves.

**Figure 2:**
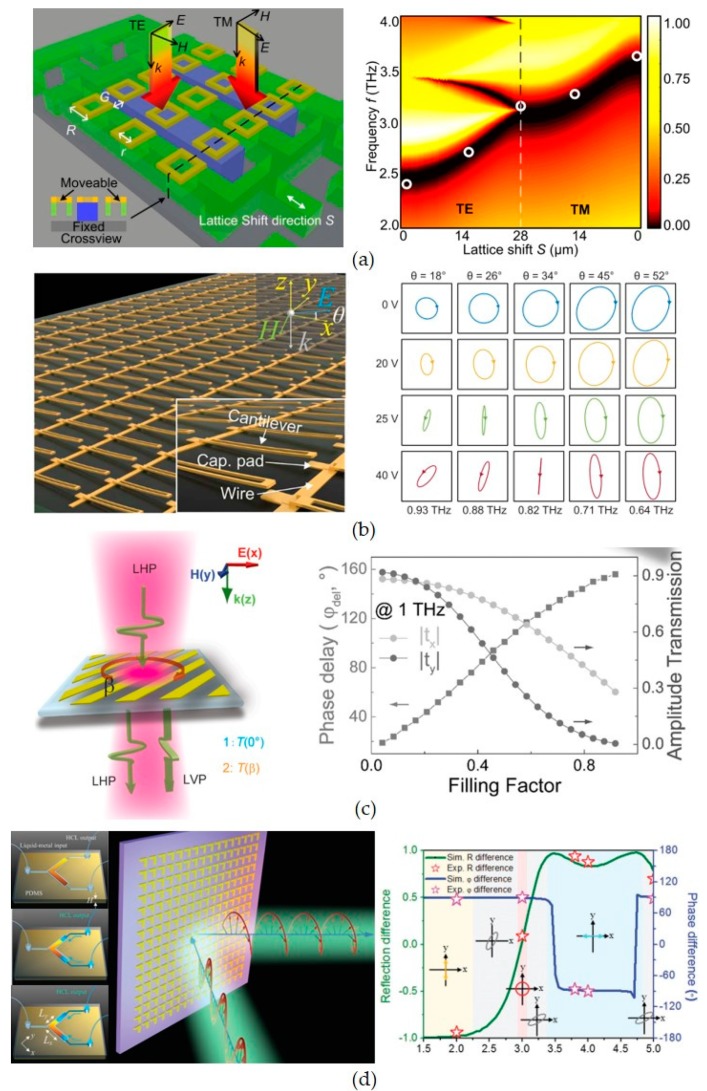
MEMS metasurfaces for polarization control. (**a**) Polarization dependent state tuning via symmetry breaking of metasurface lattice; (**b**) controllable polarizer driven by electrostatic force; (**c**) metasurface polarizer with flexible substrate; (**d**) controllable polarizer. Figure 2a–d are reprinted with permission from [64] copyright by AIP, 2011, [64] copyright by OSA Publishing, 2018, [66] copyright by Wiley Online Library, 2015 and [67] copyright by Wiley Online Library, 2017, respectively.

Tuning the symmetry of MEMS metasurface atoms is first demonstrated using electrostatic MEMS actuators [63], which are then applied to controllable polarizers. Figure 2a,b show the controllable polarizers driven by electrostatic forces realized by intra- and inner-atom structural reconfigurations, respectively [64,65], which prove the possibilities of EM wave polarization states control by changing the lattice or metamolecule symmetries. Figure 2c shows the flexible metasurface, which is tuned by MEMS actuation [66]. Figure 2d shows controllable polarizer driven by microfluidic systems [67]. The resonances induced by two orthogonal linear polarization states are coupled to each other by an ‘L’ shaped liquid metal resonator. The polarization states of the reflected EM waves are dependent on the beam lengths of ‘L’ resonator, which is controlled by the microfluidic pumps. In this way, a broadband and tunable polarization converter is realized by microfluidic metasurfaces. Recently, decoupled tuning of two orthogonal polarization states is realized using metasurfaces driven by microfluidic system [68].

### 2.2. Controllable Absorption and Emission

**Figure 3:**
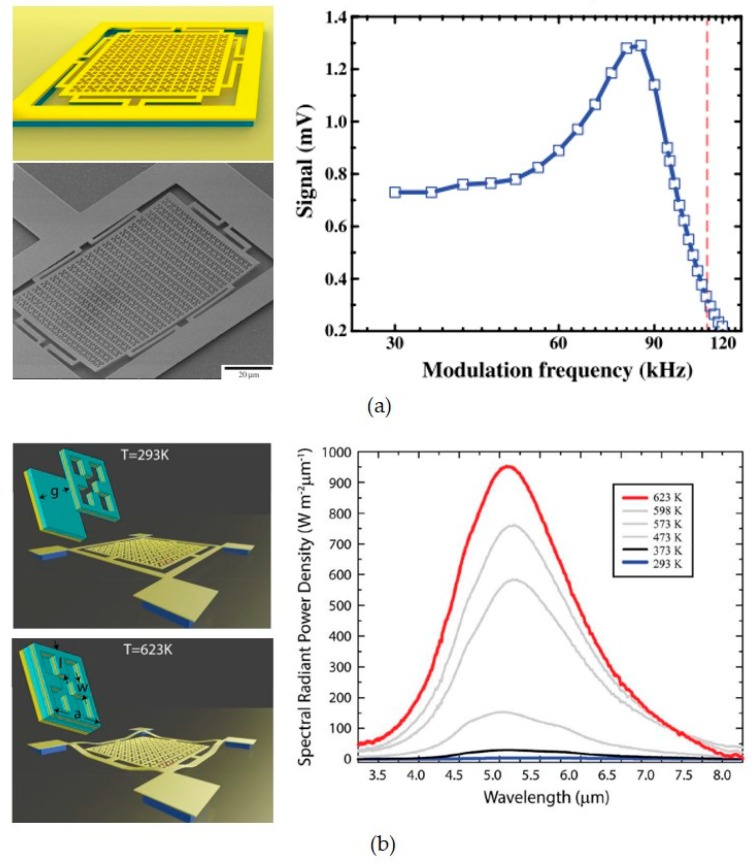
MEMS metasurfaces for controllable emission. (**a**) Controllable emitter driven by electrostatic force with maximum modulation speed of 110 KHz, the insets show schematic (up) and Scanning electron microscope (SEM) graph (down) for the emitter; (**b**) controllable emitter driven by thermo-mechanical actuation, the inserts show schematics of the emitter. Figure 3a,b are reprinted with permission from [37] copyright by OSA Publishing, 2017, and [38] copyright by Wiley Online Library, 2016, respectively.

The absorption and emission properties of metasurfaces can be controlled via MEMS actuation with KHz modulation speed. Most metasurface absorbers and emitters are multilayered structures, which are highly sensitive to the coupling between each layers. Therefore, inter-atom structural reconfigurations are applied to both controllable absorber and emitter, which work at infrared frequency region. Figure 3a,b show the MEMS metasurface emitters driven by electrostatic force [37] and thermos-actuation [38], respectively. The modulation speed can go up to 100 KHz capped by the emission efficiency, which is fast enough for most applications of controllable emitters. In the meantime, MEMS actuation does not induce extra ohmic losses or any material properties change to the emitters. Recently inner-atom structural reconfiguration is realized using microfluidic system to change the absorption band of a metasurface absorber by tuning the resonance frequencies of meta-atoms on the top layer [69,70]. The tunable absorbers based on inner-atom structural reconfiguration are demonstrated working at GHz and THz region. The metamolecules are tuned by either changing the geometry or the refractive index of the materials.

### 2.3. Reconfigurable Wavefront Manipulation

Wavefront manipulation is one of the blooming areas of metasurface research due to the promising applications in beam steering [71,72,73,74], flat lens [75,76,77,78,79,80,81], and hologram [82,83,84] etc. The dynamic tuning of wavefront manipulation is essential for many applications. For example, dynamic beam steering is the key to realize practical devices such as radar, lidar, and beam tracking devices where the tuning speed is vital, which makes MEMS metasurfaces less competitive to ultrafast metasurfaces based on material properties. However, the large tuning ranges of MEMS metasurfaces, e.g., 2-π phase change for each meta-atom, are also important for wavefront manipulations. More importantly, individually tuning of each meta-atom is also important for the reconfiguration of the wavefront. The first demonstration of individually control of meta-atoms in a 60 × 60 array is realized using metasurface driven by microfluidic system [52].

**Figure 4:**
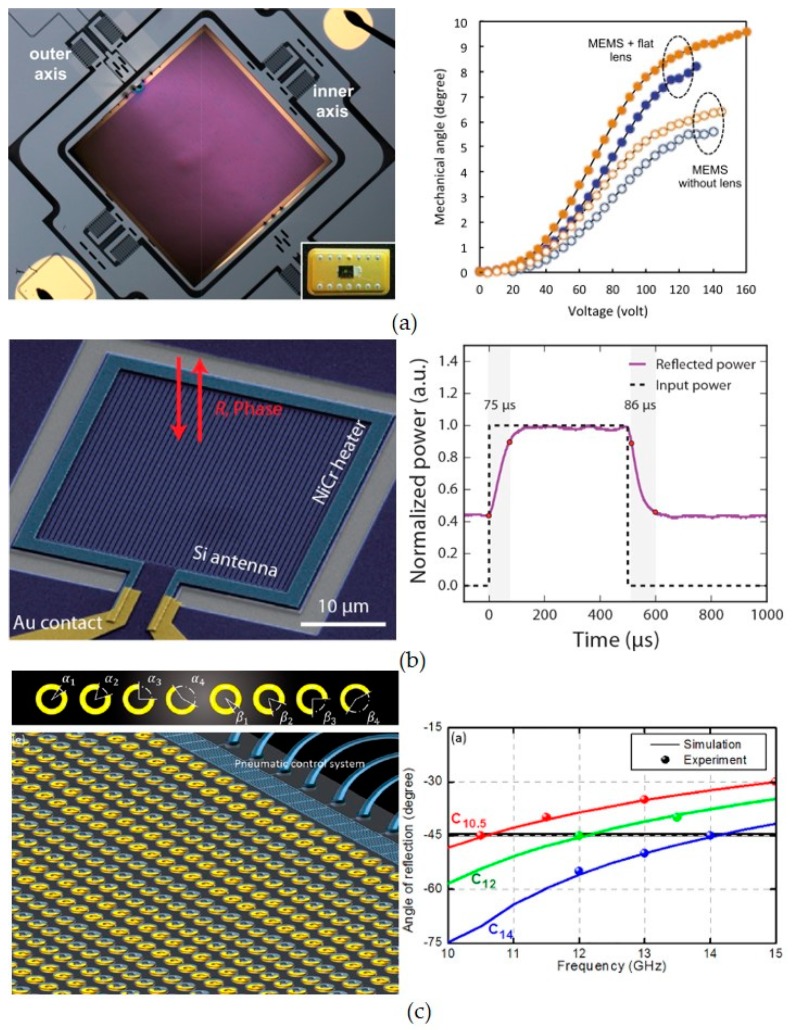
Dynamic beam steering based on MEMS metasurfaces and spatial light modulator (SLM). (**a**) Beam steering driven by electrostatic force, (**b**) SLM driven by thermos-optical effect; (**c**) metasurface beam steering driven by microfluidic system. Figure 4a–c are reprinted with permission from [85] copyright by AIP Publishing, 2018, [86] copyright by ACS Publishing, 2018, and [87] copyright by AIP Publishing, 2017, respectively.

Figure 4a–c show the dynamic beam steering driven by electrostatic force [85], thermo-optical effect [86] and microfluidic pressure pump [87], respectively. Figure 4a shows the beam steering by rotating the metasurface lens with out-of-plane MEMS actuation. It is a very robust way to have dynamic beam steering by rotating the reflection mirrors, which have been used in many commercial designs. The light-weighted metasurface lenses have higher mechanical resonance frequencies, which have fast tuning speed compared with the bulk mirrors. However, the out-of-plane actuations are not stable due to the mechanical torque introduced by the tuning process. The mechanical actuators have to be carefully designed for ultra-thin metasurface lenses. Figure 4b shows an infrared spatial light modulator (SLM) realized by thermally changing the refractive index of the material. Spatial light modulators are most commonly used devices for wavefront manipulation. However, their pixel sizes are larger than the wavelength, which inevitably affect their performances on wavefront manipulation. For example, the aberrations of the SLM have to be carefully designed for flat lens applications. The tuning speed of both MEMS metasurfaces and thermos-optical effect are in KHz region. Figure 4c shows the beam steering based on individually tuning of each meta-atoms, which is more flexible in changing the functionalities—e.g., from beam steering to dispersion compensation—but has much slower tuning speed.

**Figure 5:**
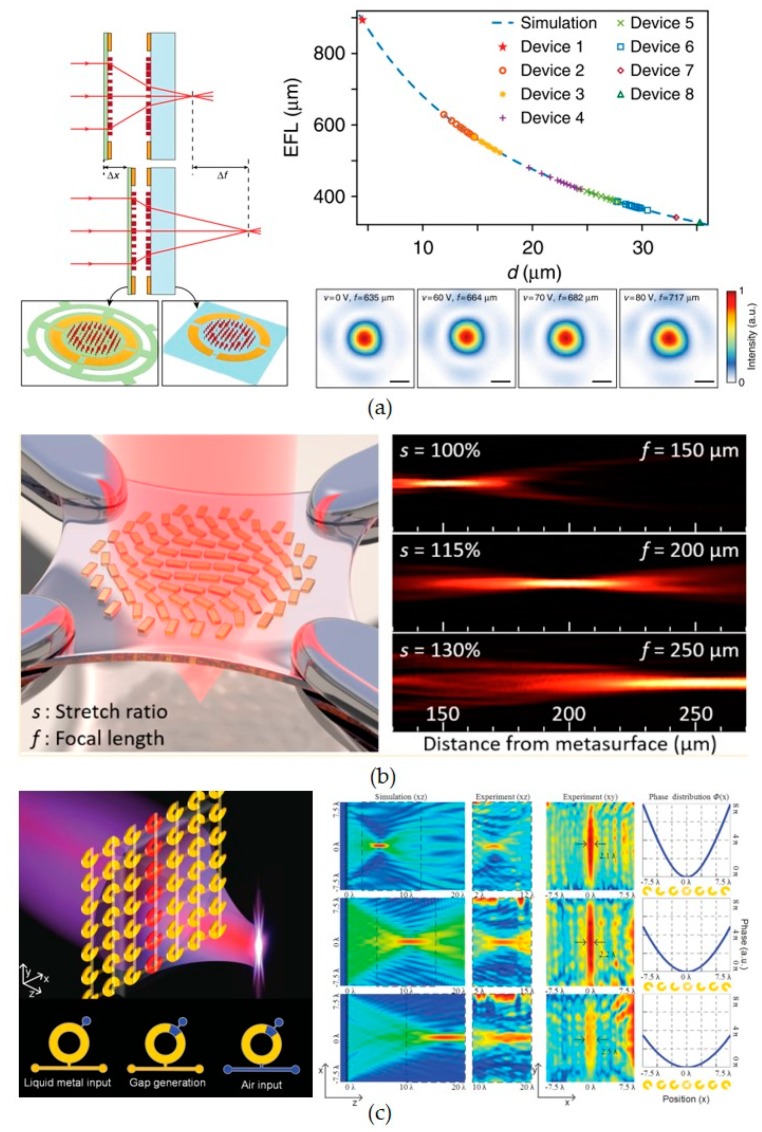
Tunable lens based on MEMS and microfluidic metasurfaces. (**a**) Tunable lens based on MEMS metasurface doublets; (**b**) tunable lens based on flexible substrate; (**c**) microfluidic metasurface tunable lens. Figure 5a–c are reprinted with permission from [88] copyright by Nature Publishing, 2018, [90] copyright by ACS, 2016, and [52] copyright by Wiley Online Library, 2015, respectively.

Unlike beam steering, KHz tuning speed is fast enough for most tunable lens applications. Recently, pioneer works on MEMS metasurface lenses show great potential on realization of on-chip fast tunable lenses. Figure 5 shows tunable metasurface lens based on MEMS and microfluidic technologies. Figure 5a shows a metasurface doublets with tunable focal length, which is controlled by changing the relative positions of two pieces of metasurface lenses. Out-of-plane MEMS actuation is also required in the mechanical design, which is similar to reference [85]. The metasurface doublets [88] are demonstrated to have 4.2 KHz modulation speed, which is thousands of times faster than its counterparts using traditional lens group [89]. Tunable metasurfaces based on flexible substrate are also demonstrated in [90]. The focal length is tuned by changing the spatial distribution of each metamolecules anchored on a flexible substrate. The aberration of the lens can be well maintained during the substrate expansion. Recently, this idea is realized using MEMS technology with better controllability [75]. However, the choice of the substrate material is very tricky to maintain the performance of the lens during the tuning process.

On the other hand, tunable metasurface lens based on microfluidic system is demonstrated at GHz region with tuning speed around 1 Hz as shown in Figure 5c. The focal length of microfluidic metasurface lens is controlled by changing the geometry of each metamolecule formed by liquid metal in microfluidic channels. A complex microfluidic control system is designed to individually tune the gaps and orientations of every metamolecules with air pressure. The metamolecules are split ring structures with gap openings orientated along either 45° or 315° to cover the 2-π phase change. The width and orientation of gap openings are tuned by the microfluidic valves. In this way, phase change of each metamolecule is individually controlled and the spatial phase change profile can be controlled at will. This metasurface is called random access reconfigurable metamaterials (RARM), which shows great potential on function switchable and adaptive metasurface devices. The major drawback is the tuning speed of RARM, which is around 1 Hz or even lower. However, the tuning speed of RARM can be greatly improved by surface treatment of the polydimethylsiloxane (PDMS) channel.

### 2.4. Fabrication Methods of MEMS and Microfluidic Metasurfaces

A high throughput and cost effective fabrication process is the key for practical applications of MEMS metasurface devices. However, metasurfaces made of subwavelength metamolecules often require high resolution fabrication technic—e.g., focus iron beam (FIB) and electron-beam lithography (EBL)—while MEMS control systems can be realized by UV lithography [91,92,93,94]. It is very difficult to have a standard process for both metamolecules and MEMS systems unless the sizes of metamolecules are big enough to work at THz or GHz region. Here, the fabrication process flows of THz MEMS metasurfaces and microfluidic metasurfaces are briefly discussed.

**Figure 6:**
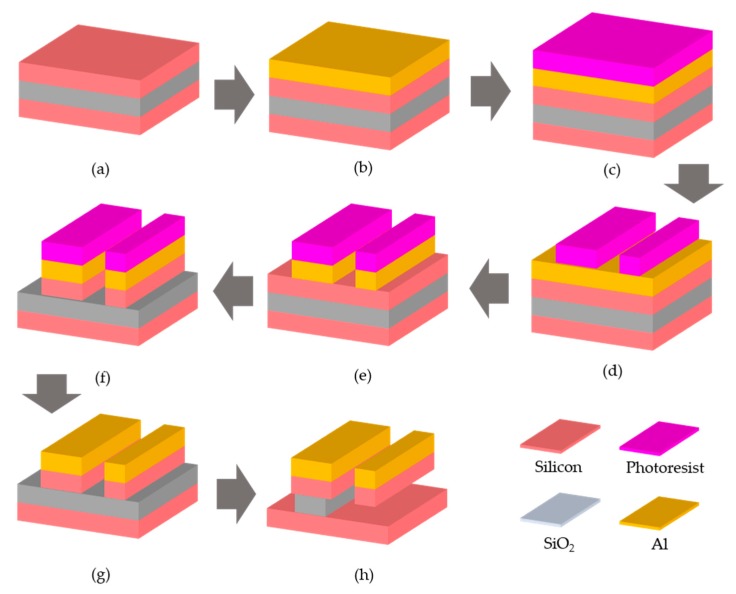
Fabrication process flow of a MEMS metasurface driven by electrostatic force, which is working at THz region. The metasurface and MEMS actuators are fabricated using the same deep reactive iron etching (DRIE) process. The movable structures are dependent on the release processes, including the chemical solution used and the control of the release time.

Figure 6 shows the fabrication process flow of a THz MEMS metasurface driven by electrostatic force [58]. The fabrication process has five main steps including aluminum sputtering, photo resist spin-off and developing, aluminum lam etching, silicon deep reactive iron etching (DRIE), and controlled wet releasing. The inserts show the materials used in the fabrication processes, which are represented by different colors.

Here, aluminum is chosen as the material for the metal structures of the metasurface, which is designed to work at THz region. The substrate is a silicon on insulator (SOI) wafer for MEMS structures, which is shown in Figure 6a. Firstly, an aluminum film is sputtered on the SOI substrate and forms a four-layer structure, which includes, from top to bottom, a 200-nm aluminum film, a 75-μm single crystal silicon layer, a 200-nm silicon dioxide layer, and a 600-μm single crystal silicon substrate as shown in Figure 6b. The first silicon layer serves as the supporting layer for metasurface structures, which are partially released from the silicon substrate. The silicon dioxide layer is used as the stop line for the DRIE process. The thickness of each layer can be chosen based on the design of MEMS metasrufaces.

The second step shown in Figure 6c,d is to pattern a photoresist (PR) layer and define the metal structures of the metamolecules. Two different masks are applied during the fabrication process. One is to pattern the PR layer for the aluminum structures and the dicing lines. The other one is for a silicon-dioxide thin film, called hard mask layer, which is applied to protect other MEMS structures during the lam etching (Figure 6e) of the metal part. In this way, the metamolecule structures made of aluminum are patterned on the top of SOI wafer. The minimum feature size of the metamolecule structures are limited by the photo mask used to define the PR pattern, which is 1 μm in [58].

Then another PR layer is spin-coated on the top of SOI wafer with aluminum patterns and another photo mask with minimum feature size of 3 μm is applied to define the MEMS structures, such as supporting frames, comb drives, and isolated substrate for metamolecules as shown in Figure 6f. The DRIE process is applied to etch the MEMS structures on the top silicon layer of SOI wafer, which is a standard process for most silicon photonic devices. Also, a sidewall passivation process is applied to ensure the uniformity of the side wall of MEMS structures. Here, some parts of the aluminum patterns have larger silicon substrate, which requires more releasing time. In the meantime, some aluminum parts have very small silicon substrates, which can be released in a relatively shorter releasing time.

The metamolecules are composed of two different parts. One is connected to and can be driven by the MEMS actuator, which is called movable part. The other one is anchored on an isolated substrate, which is called fixed part. The realization of the fixed and movable parts of the metamolecules are highly dependent on the wet releasing process after DRIE, which is shown in Figure 6g,h. The releasing time is affected by many fabrication parameters including the size and spacing of the silicon microstructures, the chemical and concentration of the release solution etc. Here, buffered oxide etchant (BOE) is applied to release the MEMS comb drivers and the movable parts of the metamolecules. However, the fixed parts of the metamolecules are under the same wet releasing process as well. The movable parts of the metamolecules are patterned on a silicon frame with 3 μm in width, which is similar to those of the MEMS comb driver structures. In the meantime, the fixed parts are patterned on isolated substrates with tens of microns in width, which have much longer releasing time than those of the silicon frame and MEMS structures. During the fabrication process, the releasing time is finely controlled so that the movable parts of the metamolecules are released while the substrates of fixed parts are still anchored on the silicon dioxide layer, which is stick to the backside layer of 600-μm silicon.

To ensure the ohmic contact of the movable and fixed parts of metamolecules, a shadow mask is applied to deposit a 200-nm aluminium film by a sputtering system on the side walls of the gaps in between. Also, the 600-μm silicon back side layer is thinned to 100 μm to minimize the Fabry–Pérot resonance observed in experiment. These two steps are optional, which is not included in Figure 6. It should be pointed out that much finer metal structures can be patterned on or transferred to the MEMS structures, which can be used for inter-atom structural reconfiguration working at higher frequencies.

**Figure 7:**
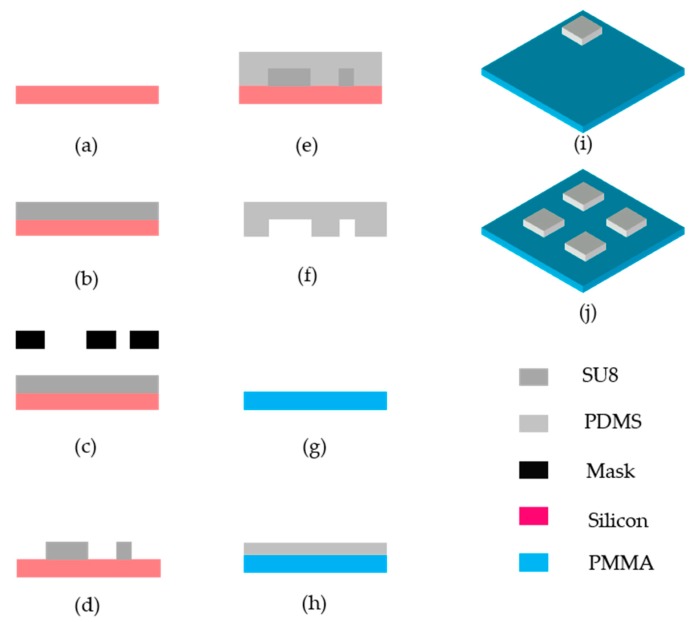
Soft lithography process flow of microfluidic metasurfaces working at GHz region. The control system and the microfluidic channels hosting the liquid metal droplets are fabricated using the same process. The large array of metamolecules is realized by multi-sample stitching technique.

The microfluidic metasurfaces are fabricated using soft lithography processes [95,96,97], which are shown in Figure 7. The inserts show different materials used in [52]. The fabrication processes can be divided into three main steps, which are master preparation (Figure 7a–d), microfluidic channel molding (Figure 7e–h), and multi-sample stitching (Figure 7i–j). Master preparation is to fabricate an inverse structure of designed microfluidic channels using different materials including silicon, SU-8 photoresist, polymethylmethacrylate (PMMA), etc. Silicon substrate can be patterned by UV-lithography with large range of feature sizes from sub-micron to tens of microns. However, the silicon substrate need special surface treatment to be used as the master of liquid plastic materials such as polydimethylsiloxane (PDMS). Therefore, it is only used for microfluidic channels with minimum feature size less than 10 μm. SU-8 photo resist is often used as the master of PDMS channels with the feature sizes ranging from 10s to 100s μm, which can be formed using soft lithography processes. For larger channels, the master can be formed using PMMA and micromachining technology, which is more cost effective than lithography.

Figure 7 shows the master preparation based on SU-8 photoresist. Firstly, the SU-8 photoresist is spin coated on a silicon substrate as shown in Figure 7b. Then the SU-8 is soft-baked by using a hotplate at 65 °C for 10 min and at 95 °C for 25 min. The designed microfluidic structures are transferred to SU-8 photoresist by using the plastic mask and UV exposure, as shown in Figure 7c. After that, the sample is put into the oven at 65 °C for 3 min, and then, an oven at 95 °C for 10 min to enhance the combination of the SU-8 and the silicon substrate, which is called post bake. The inverted patterns of microfluidic channels are formed by using SU-8 photoresist after development. Afterwards, the patterned sample is put into the oven for several hours to ensure the combination of SU-8 photoresist and silicon substrate.

Once the master is ready, the PDMS is mounted onto the sample and solidified, as shown in Figure 7e. Typically, an oven is used to hasten the process, which can be controlled by the environmental temperature. Then, the structured PDMS layer is peeled off, as shown in Figure 7f, from the sample and bonded with polymer substrate to form microfluidic channels of the metasurfaces. The polymer substrates are often coated with a thin layer of PDMS to enhance the combination of microfluidic channel layer and substrate so that the channels can withstand large inject pressure.

The lithography technique can only handle a limited size of substrate. However, large sample size is often required for GHz devices. Therefore, multi-sample stitching technique is developed for large sample fabrication, as shown in Figure 7j. Each part of the sample is fabricated using the soft-lithography process individually and then stitched together on a large substrate to form the whole sample as reported in [58].

### 2.5. Summary of MEMS and Microfluidic Metasurfaces

Table 1 summarizes the applications of MEMS metasurfaces based on operation band, tuning speed, and mechanism. During the last 10 years, vast applications have been demonstrated using MEMS metasurfaces, including lenses, filters, polarizers, optical switches, absorbers, emitters, and beam steering devices. Most MEMS metasurfaces are tuned by mechanical actuations such as electrostatic actuation, thermos-actuation, microfluidic pressure actuation, etc. Generally speaking, MEMS actuators have minimum feature size of several microns, which is similar to the metamolecules at sizes of tens of microns. Therefore, MEMS metasurfaces are demonstrated to work at GHz, THz, and infrared region due to the fabrication compatibility and feasibility. However, it is possible for intra-atom structural reconfiguration to work at visible band with smart design. The tuning speeds for MEMS metasurfaces are from KHz to MHz, which is sufficient for most applications, such as tunable lens, controllable absorber, and polarizer. Microfluidic metasurfaces have very slow tuning speed, which is limited by current technology. For mechanical actuation, small mass in general results in fast tuning speed, which is capped by the mechanical resonance frequency of the system. On the other hand, the size and mass of metamolecules are inversely proportional to their working frequency. It can be expected that, with the development of NEMS and microfluidic technology, metasurfaces driven by mechanical actuation will be demonstrated working in the visible frequency region with tuning speeds at MHz or above.

## 3. Tunable Lens Based on MEMS Metasurfaces

The research on metasurface lenses are focused on surpassing the performance of traditional lenses. Many efforts have been made to eliminate chromatic aberration so that the metasurface lenses can work on a broad frequency band [92,98,99,100,101]. On the other hand, the focus adjustable lenses have been the subject of traditional lens researches for a number of years. The tuning speeds of most traditional lenses are around tens of Hz due to their bulk size and large mass, which hump the applications of tunable lenses. For example, most visual tracking systems rely on software zooming/deburr to track targets with high moving speed. The bulk sizes of tunable lenses also limit their applications on compact devices, such as smart phones. On the other hand, the compact and light-weighted metasurface lenses are good candidates for fast tunable lenses via mechanical actuations, which has been demonstrated with tuning speed up to KHz.

The focus adjustable lenses can be divided into three categories. Firstly, tunable focus can be realized by tuning the relative positions of lenses in a group, e.g., the doublets [102], Alvarez lens [89], etc. The tunable lens groups are widely used in cameras and microscopes, which are typically bulky. Secondly, tunable lenses can be realized by changing the curvature of curved lens [103,104]. Those tunable lenses are made of soft materials—i.e., polymer and liquid—which can be deformed by mechanical actuation. Therefore, the choices of lens materials are quite limited, especially for those lenses working at THz and infrared region. Thirdly, the focal length can be tuned by changing the refractive indexes of the lenses or the surrounding media [105]. For example, liquid crystal lenses can be tuned by changing the refractive indexes using external voltage, thermos-effect or even photo luminance. However, it is very difficult to maintain the desired refractive index distribution during the tuning process. It should be point out that tuning the abrupt phase change of metamolecules also can control the focal length of the metasurface lens [52], which is similar to the refractive indexes change of traditional lenses.

**Figure 8:**
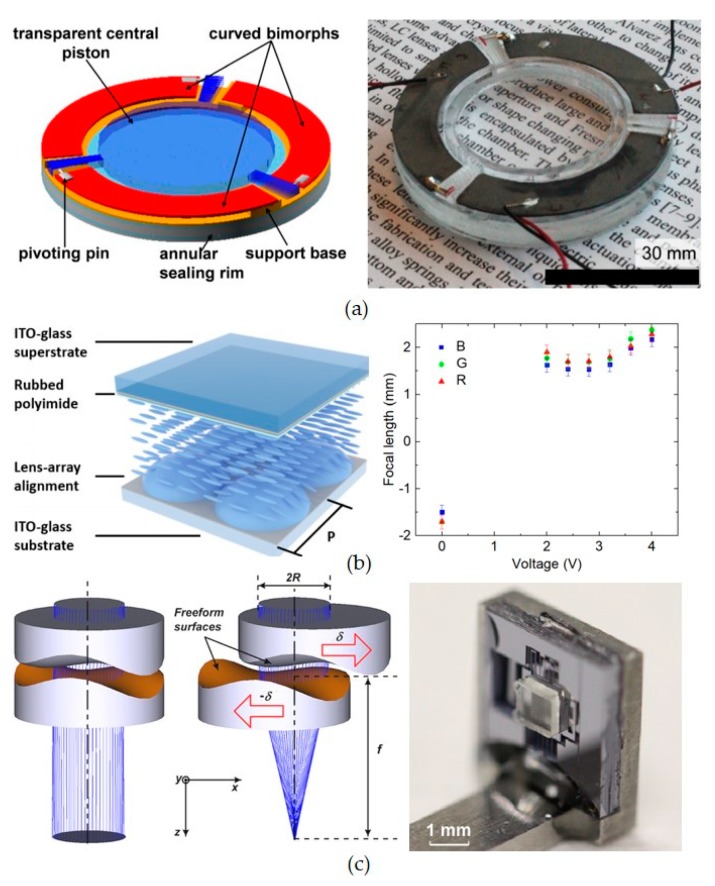
Tunable lenses realized by various approaches. (**a**) Curvature change of lens with soft materials; (**b**) tunable lens realized by changing the refractive index of materials; (**c**) adjustable-focus endoscope realized by lens group based on Alvarez principle. Figure 8a–c are reprinted with permission from [106] copyright by OSA Publishing, 2017, [107] copyright by OSA Publishing, 2018, and [89] copyright by OSA Publishing, 2015, respectively.

Figure 8a shows the tunable lens based on the curvature change driven by a piezoelectric actuator, which can be designed to have fast actuation speed up to MHz [106]. The diameter of the tunable lens is 32 mm with optical power range of 5.6 diopter, and electrical power consumption less than 20 mW. The tunable lens is made of liquid material (glycerol) with the thickness of 8.4 mm and weight of 14.4 g, which are 10^3^ times larger than a typical metasurface lens. The measured resonance frequency is only 70 Hz due to the bulk size and large weight of liquid lens, which is the bottleneck of the tuning speed.

Figure 8b shows a tunable lens array realized by changing the refractive index of the surrounding media—i.e., liquid crystal—which has fast tuning speed around KHz [107]. A 16 × 16 micro lenses array made of polymer is demonstrated to have focal length tuning from −2 mm to 2 mm. In this work, 3D fabrication technology is required for micro lenses array. This method is also applied to metasurface to have the tunable EM properties. The tuning range is limited by the refractive index change of liquid crystal.

Figure 8c shows the adjustable focal lens realized by traditional lens group. The lens group is composed of two freeform lenses, which are governed by 6-degree polynomials and optimized by using ray tracing simulation method. In experiment, the optical power of the lens group is tuned from approximately 135 diopters to 205 diopters. However, Similar to Figure 8a, the mechanical actuation limits the tuning speed of bulk lenses.

**Figure 9:**
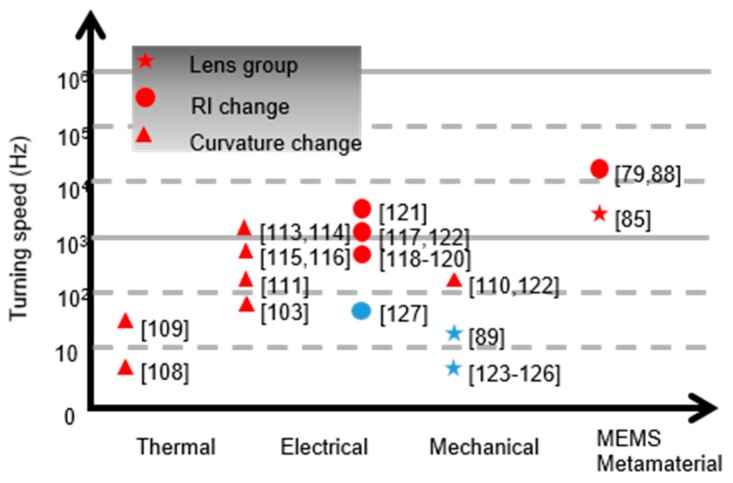
Summary of tunable lens by the tuning speeds and mechanisms. The red symbols represent tunable lenses, which can be applied to integrated systems. The blue symbols represent bulk and heavy lenses.

Figure 9 shows a summary of traditional and MEMS metasurface lens. The star, circular and triangle symbols represent tunable lenses based on lens groups, refractive index (RI) changes and lens curvature deformations. The red color represents compact lenses, which can be applied to integrated systems while the blue color represents bulk and heavy lenses. Pioneer works on MEMS metasurface lenses are listed on the right column. Indeed, the tuning mechanisms—including thermal [108,109], mechanical [110], and electrical tuning [111]—affect the speed of the tunable lenses. Most tunable lenses are realized by either electrical or mechanical tuning [112,113,114,115,116,117,118,119,120,121,122,123,124,125,126,127]. Therefore, the tuning speed is, in most cases, determined by the size and weight of the lenses. Take mechanical actuation as an example, the smaller weight results in higher resonance frequency of the mechanical system, which is the bottleneck of the actuation speed with large displacement. The metasurface lenses are more likely to have fast speed based on current tuning mechanisms due to the small size and light weight, which have recently been demonstrated to have KHz tuning speed. It can be expected that the MEMS metasurface lens can work at higher frequency (visible range) and faster tuning speed (MHz) with the development on MEMS/NEMS technologies, which make them good candidates for fast and compact imaging systems.

## 4. Summary and Outlook

Research on metasurfaces has now been shifted from fundamental studies to practical applications, of which MEMS metasurfaces have long been considered as a supplement to active metasurfaces when fast tuning speed is not a necessity. During the last decade, vast applications have been demonstrated using MEMS metasurfaces which proved to be an important approach to the realization of densely integrated tunable EM devices. The modulation speed of most MEMS metasurfaces is at KHz, which is hundreds of times faster than current tuning speed of tunable lens. Now the demand for fast tunable lens is increasing due to the development for smart cameras and machine vision. However, the state-of-art technology has limited tuning speed, which is capped at tens of Hz. On the other hand, MEMS metasurfaces have become a promising candidate for fast focus tuning due to their small size, light weight, capability of dense integration, and compatibility of cost effective fabrication process. It can be expected that MEMS tunable lenses will make a new impact on the metalens research community with fast tuning speed and adjustable functionalities.

## Figures and Tables

**Table 1:** Summary of the applications of microelectromechanical systems (MEMS) metasurfaces based on modulation mechanism, operation band, and tuning speed.

	Applications	Modulation Mechanism	Operation Band	Speed	References
Frequency, amplitude and polarization state	Filter	Electrostatic actuation	THz	KHz	[44,45]
		Thermo-actuation	THz	KHz	[61]
	Polarizer	Electrostatic actuation	THz	KHz	[63,64,65]
		Microfluidic pressure actuation	GHz	Hz	[67,68]
	Switch	Electrostatic actuation	NIR	KHz to MHz	[5,25]
		Thermo-optical effect	IR	KHz	[49]
Absorption and emission	Absorber	Electrostatic actuation	THz	KHz	[7]
		Thermo-actuation	THz	KHz	[42,59]
		Microfluidic pressure actuation	GHz to THz	Hz	[69,70]
	Emitter	Electrostatic actuation	IR	KHz to MHz	[37]
		Thermo-actuation	IR	KHz	[38]
Wavefront manipulation	Beam Steering	Electrostatic actuation	GHz	Not reported	[71,72,85]
		Thermo-optical effect	MIR	KHz	[86]
		Microfluidic pressure actuation	GHz	Hz	[74,87]
	Lens	Electrostatic actuation	GHz	Not reported	[79]
		Microfluidic pressure actuation	GHz	Hz	[52,80]
